# Síndrome de Marfan, Cardiomiopatia Hipertrófica e QT Longo, uma Associação Rara como Causa de Morte Súbita

**DOI:** 10.36660/abc.20230489

**Published:** 2024-06-26

**Authors:** Diana Carvalho, Simão Carvalho, Adriana Pacheco, Carlos Costa, Pedro Carvalho, Raquel Ferreira, Ana Briosa

**Affiliations:** 1 Centro Hospitalar Baixo Vouga Aveiro Portugal Serviço de Cardiologia – Centro Hospitalar Baixo Vouga, Aveiro – Portugal; 2 Centro Hospitalar Tâmega e Sousa Penafiel Portugal Serviço de Cardiologia – Centro Hospitalar Tâmega e Sousa, Penafiel – Portugal

**Keywords:** Arritmias Cardíacas, Cardiomiopatia Hipertrófica, Síndrome de Marfan, Prolapso da Valva Mitral, Morte Súbita Cardíaca

## Introdução

A síndrome de Marfan (SM) é uma doença sistêmica do tecido conjuntivo com transmissão autossômica dominante, geralmente associada a uma mutação no gene da fibrilina 1 (FBN1). A prevalência estimada é de 6,5/100.0001. De acordo com os critérios revisados de Ghent, a mutação no gene FBN1, a ectopia do cristalino e a dilatação da raiz da aorta são os fatores-chave para o diagnóstico de SM.^1^^,^^2^ A esperança de vida é essencialmente determinada pelas complicações cardiovasculares, particularmente a aortopatia.

## Relato de Caso

**Figura 1:**
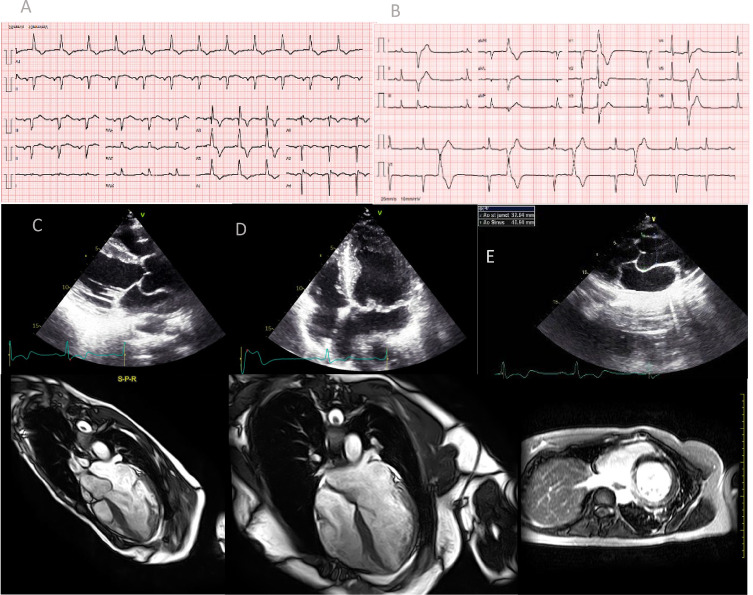
– Eletrocardiograma evidenciando intervalo QT prolongado (A) e bigeminismo ventricular (B). Ecocardiograma demonstrando hipertrofia assimétrica, prolapso mitral e raiz aórtica de dimensões normais (C, D, E). RM com hipertrofia assimétrica (F, G) e realce miocárdico tardio nas paredes inferior e septal (H).

Os autores descrevem o caso de uma mulher de 31 anos, com forte história familiar de morte súbita inexplicável (pai e três irmãos morreram entre os 20-30 anos). Foi internada após reanimação de parada cardíaca súbita em ritmo de fibrilação ventricular em outubro de 2018. O eletrocardiograma de admissão mostrou ritmo sinusal, ondas T negativas na parede inferior e intervalo QT corrigido prolongado de 497 mseg (Figura 1A). Na monitorização eletrocardiográfica, a paciente apresentava frequentes contrações ventriculares prematuras e períodos de bigeminismo ventricular (Figura 1B). A gasometria e os exames de sangue na admissão não apresentaram alterações significativas. O ecocardiograma transtorácico mostrou ventrículo esquerdo com dimensões normais e hipertrofia assimétrica do septo interventricular (espessura máxima de 17 mm no septo anterior), sem obstrução da via de saída do ventrículo esquerdo e sem alterações de motilidade regional. A função sistólica biventricular estava preservada; havia evidência de prolapso da valva mitral (PVM) levando a regurgitação mitral moderada; o átrio esquerdo estava moderadamente dilatado; raiz aórtica e aorta ascendente apresentavam dimensões normais e ausência de retalho intimal (Figuras 1C, D e E). A angiografia coronária excluiu doença coronária obstrutiva. A paciente foi extubada sem déficits neurológicos significativos e internado no Serviço de Cardiologia.

Fenotipicamente, a paciente era esbelta, com membros longos, aracnodactilia e deformidade em forma de V no palato. Ela também apresentava aumento da elasticidade articular, sinal positivo no punho e polegar, assimetria torácica, enoftalmia, fissuras palpebrais inclinadas para baixo, retrognatia e estrias cutâneas. O exame oftalmológico mostrou miopia e subluxação do cristalino (escore sistêmico de 9 pontos para Síndrome de Marfan).

A ressonância magnética (RM) cardíaca mostrou hipertrofia assimétrica do septo e da parede anterior médio-apical e adelgaçamento da parede póstero-lateral. A RM também apresentou realce tardio miocárdico localizado na parede média inferior inferoseptal e basal e realce tardio mais heterogêneo no septo médio (Figuras 1F, G e H). A aorta apresentava ectasia dos seios de Valsalva (36 mm; escore Z 1,92).

O estudo genético foi negativo para cardiomiopatia hipertrófica (CMH) e síndrome do QT longo, mas identificou a variante c.4099T>C (p.Cys1367Arg) em heterozigosidade do gene FBN1, considerada patogênica. O painel genético da CMH avaliado foi: ACTC1 (NM_005159.4), DES (NM_001927.3), GLA (NM_000169.2), LAMP2 (NM_001122606.1), MYBPC3 (NM_000256.3), MYH7 (NM_000257.2), MYL2 (NM_000432.3), MYL3 (NM_000258.2), PLN (NM_002667.3), PRKAG2 (NM_016203.3), PTPN11 (NM_002834.3), TNNC1 (NM_003280.2), TNNI3 (NM_000363.4), TNNT2 (NM_000364.2), TPM1 (NM_001018005.1), TTR (NM_000371.3) e TCAP (NM_003673.3).

Um cardioversor-desfibrilador implantável subcutâneo (CDI-s) foi implantado para prevenção secundária antes da alta.

Após um período de acompanhamento de dois anos, o paciente desenvolveu insuficiência cardíaca com regurgitação mitral grave devido ao prolapso de ambos os folhetos da valva mitral (predominantemente o folheto anterior). Apresentava fração de ejeção do ventrículo esquerdo preservada (65%). Na monitorização eletrocardiográfica de 24 horas, apresentava contrações ventriculares prematuras polimórficas muito frequentes e sintomáticas (23322 em 24 horas), com períodos de taquicardia ventricular não sustentada, que não melhoraram após a introdução de amiodarona. A paciente foi submetida a valvoplastia mitral incluindo ressecção de P2 e implante de anel mitral e neocordas. Após a cirurgia, a carga de arritmia ventricular foi marcadamente reduzida.

Nenhuma dilatação aórtica adicional ou terapias com CDI-s durante o acompanhamento.

## Discussão

Segundo os critérios de Ghent II, a presença de ectopia lentis e a mutação no gene FBN1 sugerem o diagnóstico de SM, considerando história familiar sugestiva. Além disso, a presença de características sistêmicas também corrobora o diagnóstico de SM (pontuação de 8 pontos segundo os critérios de Ghent II).^2^ A dilatação aórtica está presente em cerca de 75,8% dos pacientes com SM.^3^^,^^4^ Neste caso, a ausência de dilatação aórtica é um fator de confusão que pode dificultar o diagnóstico.

A ocorrência de fibrilação ventricular, neste caso específico, poderia ser decorrente de diversos fatores apresentados pelo paciente na admissão, especificamente a presença de PVM, prolongamento do intervalo QT e documentação de hipertrofia ventricular assimétrica com carga cicatricial na ressonância magnética.

As arritmias são relativamente comuns na SM, sendo a fibrilação atrial a mais frequente (14,8% dos pacientes).^5^ Alguns estudos sugerem que a ocorrência de morte cardíaca súbita de causa presumivelmente arrítmica pode ocorrer em até 4% dos pacientes. Porém, trata-se de séries com número pequeno de pacientes e o evento arrítmico esteve associado à maior dilatação do ventrículo esquerdo.^6^^,^^7^ Num estudo que incluiu 12.079 pacientes com SM, a fibrilação ventricular é responsável por 0,5% de todas as internações hospitalares por doenças cardíacas e 0,2% de todas as internações hospitalares.^5^

Em pacientes com SM, o prolapso da valva mitral e a regurgitação mitral grave têm prevalência de 40% e 12%, respectivamente.^8^ Embora a associação entre PVM isolado e arritmias ventriculares não seja consensual, estudos sugerem que a coexistência de insuficiência mitral moderada se correlaciona com a presença de contrações ventriculares prematuras e taquicardia ventricular não sustentada. Contudo, não parece predizer a ocorrência de eventos arrítmicos graves.^6^^,^^7^^,^^9^ Neste paciente, a redução acentuada da carga de arritmias ventriculares após valvoplastia mitral favorece a hipótese de que a valvopatia mitral foi o principal fator que levou à fibrilação ventricular.

O prolongamento do QTc (>440 mseg) pode estar presente em até 16-20% dos pacientes com SM.^10^ Alguns estudos também demonstraram associação entre dilatação ventricular esquerda, alterações de repolarização e ocorrência de arritmias cvpventriculares.^6^^,^^7^^,^^10^ Neste caso clínico não foi documentada dilatação ventricular esquerda, o que sugere que o intervalo QT longo parece ser um preditor independente de arritmias ventriculares. Entretanto, embora a associação com a presença de CVP e TVNS pareça estabelecida, sua associação com eventos arrítmicos mais graves não parece ser bem compreendida.^7^^,^^10^

A cardiomiopatia associada à SM é uma entidade cada vez mais reconhecida, mas geralmente está relacionada com dilatação e disfunção ventricular.^3^ Nesta paciente, o fenótipo de cardiomiopatia hipertrófica pode estar relacionado à mutação no gene FNB1. No entanto, é mais provável que estejamos lidando com duas condições distintas – cardiomiopatia hipertrófica e síndrome de Marfan. A ausência de teste genético positivo para CMH não exclui o diagnóstico, pois o rendimento do teste genético é de apenas 30 a 40%.^11^ Após revisão da literatura, encontramos apenas um caso descrevendo CMH concomitante com SM. Assim, esta parece ser uma associação extremamente rara e ainda não estudada anteriormente.^12^ No caso descrito por Fujiseki et al., foi documentada hipertrofia assimétrica do septo interventricular, com relação espessura da parede septal-inferolateral de 2,5 (medida pelo modo M). No presente caso, houve assimetria mais pronunciada, com relação espessura da parede septal-inferolateral de 3,2 (medida por ressonância magnética).

Neste paciente, a combinação desses três fatores (prolapso mitral com regurgitação moderada, intervalo QT longo e CMH) pode ter sido decisiva para a ocorrência de fibrilação ventricular.

## Conclusão

Este caso relata uma apresentação atípica de SM com fibrilação ventricular, em oposição à dilatação e dissecção da aorta. Em uma paciente com ectopia lentis e mutação no gene FBN1, uma história familiar de morte súbita apoia o diagnóstico de Síndrome de Marfan na ausência de dilatação da raiz da aorta. Embora seja um assunto com crescente atenção, a evidência de arritmias malignas ainda é uma área cinzenta na SM. A dilatação ventricular esquerda e a disfunção miocárdica podem ter um papel causal, mas estas alterações não foram documentadas nesta paciente. Outras alterações, nomeadamente o QT longo e o PVM com regurgitação moderada, poderão ter contribuído para o evento arrítmico. No entanto, esta paciente também apresenta CMH, que é um fator pró-arrítmico adicional, e cuja associação com SM parece ser extremamente rara.
