# Supplementary material for: Síndrome de Marfan, Cardiomiopatia Hipertrófica e QT Longo, uma Associação Rara como Causa de Morte Súbita
Source: Arq Bras Cardiol. 2024 Jun 26;121(5):e20230489. [Article in Portuguese] doi: 10.36660/abc.20230489 (PMC12087630; doi:10.36660/abc.20230489)
Supplement: Translation

# Translation: Marfan Syndrome, Hypertrophic Cardiomyopathy And Long QT A Rare Association Causing Sudden Death

**DOI:** 10.36660/abc.20230489i

**Authors:** Diana Carvalho, Simão Carvalho, Adriana Pacheco, Carlos Costa, Pedro Carvalho, Raquel Ferreira, Ana Briosa

**Affiliations:** 1 Portugal Serviço de Cardiologia – Centro Hospitalar Baixo Vouga, Aveiro – Portugal; 2 Portugal Serviço de Cardiologia – Centro Hospitalar Tâmega e Sousa, Penafiel – Portugal

**Keywords:** Cardiac Arrhythmias, Hypertrophic Cardiomyopathy, Marfan Syndrome, Mitral Valve Prolapse, Cardiac Sudden Death

## Introduction

Marfan syndrome (MS) is a systemic disease of the connective tissue with an autosomal dominant transmission, usually associated with a mutation in the fibrillin 1 gene (FBN1). The estimated prevalence is 6.5/100,000. According to the revised Ghent criteria, mutation in the FBN1 gene, lens ectopy, and aortic root dilation are the key factors for the diagnosis of MS.^1^^,^^2^ Life expectancy is essentially determined by cardiovascular complications, particularly aortopathy.

## Case Report

The authors describe the case of a 31-year-old woman, with a heavy family history of sudden unexplained death (father and three brothers died between 20-30 years of age). She was admitted after resuscitation from sudden cardiac arrest in ventricular fibrillation rhythm in October 2018. The electrocardiogram on admission showed sinus rhythm, negative T waves in the inferior wall, and a prolonged corrected QT interval of 497 msec (Figure 1A). On electrocardiographic monitoring, the patient had frequent premature ventricular contractions and periods of ventricular bigeminy (Figure 1B). Blood gas analyses and blood tests on admission did not show significant alterations. The transthoracic echocardiogram showed a left ventricle with normal dimensions and asymmetrical hypertrophy of the interventricular septum (maximum thickness of 17 mm in the anterior septum), without left ventricular outflow tract obstruction and without regional wall motion abnormalities. Biventricular systolic function was preserved; there was evidence of mitral valve prolapse (MVP) leading to moderate mitral regurgitation; the left atrium was moderately dilated; aortic root and ascending aorta had normal dimensions and no intimal flap (Figures 1C, D and E). Coronary angiography excluded obstructive coronary disease. The patient was extubated without significant neurological deficits and admitted to the Cardiology Department.

**Figure 1:** – Electrocardiogram showing prolonged QT interval (A) and ventricular bigeminy (B). Echocardiogram showing asymmetric hypertrophy, mitral prolapse, and aortic root with normal dimensions (C, D, E). MRI with asymmetric hypertrophy (F, G) and delayed myocardial enhancement in the inferior and septal walls (H).

Phenotypically, the patient was slender, with long limbs, arachnodactyly, and a V-shaped deformity of the palate. She also had increased joint elasticity, a positive wrist and thumb sign, chest asymmetry, enophthalmos, downslanting palpebral fissures, retrognathia, and skin striae. The eye examination showed myopia and lens subluxation (9-point systemic score for Marfan Syndrome).

Cardiac magnetic resonance imaging (MRI) showed asymmetric hypertrophy of the septum and medium-apical anterior wall and thinning of the posterolateral wall. MRI also presented delayed myocardial enhancement located on the inferoseptal and basal inferior mid-wall and more heterogeneous delayed enhancement in the midseptum (Figures 1F, G, and H). The aorta presented ectasia of the sinuses of Valsalva (36 mm; Z-score 1,92).

The genetic study was negative for hypertrophic cardiomyopathy (HCM) and long QT syndrome but identified the variant c.4099T>C (p.Cys1367Arg) in heterozygosity of the FBN1 gene, considered pathogenic. The HCM genetic panel evaluated was: ACTC1 (NM_005159.4), DES (NM_001927.3), GLA (NM_000169.2), LAMP2 (NM_001122606.1), MYBPC3 (NM_000256.3), MYH7 (NM_000257.2), MYL2 (NM_000432.3), MYL3 (NM_000258.2), PLN (NM_002667.3), PRKAG2 (NM_016203.3), PTPN11 (NM_002834.3), TNNC1 (NM_003280.2), TNNI3 (NM_000363.4), TNNT2 (NM_000364.2), TPM1 (NM_001018005.1), TTR (NM_000371.3) and TCAP (NM_003673.3).

A subcutaneous implantable cardioverter-defibrillator (s-ICD) was implanted for secondary prevention before discharge.

After a two-year follow-up period, the patient developed heart failure with severe mitral regurgitation due to prolapse of both mitral valve leaflets (predominantly the anterior leaflet). She had preserved left ventricular ejection fraction (65%). On 24-hour electrocardiographic monitoring, she had very frequent and symptomatic polymorphic premature ventricular contractions (23322 in 24 hours), with periods of non-sustained ventricular tachycardia, that did not improve after the introduction of amiodarone. The patient underwent a mitral valvuloplasty including P2 resection and mitral annulus and neocords implantation. After surgery, the ventricular arrhythmia burden was markedly reduced.

No additional aortic dilation or s-ICD therapies during follow-up.

## Discussion

According to the Ghent II criteria, the presence of ectopia lentis and the mutation in the FBN1 gene suggest the diagnosis of MS, considering a suggestive family history. In addition, the presence of systemic features also corroborates the diagnosis of MS (score of 8 points according to the Ghent II criteria).^2^ Aortic dilation is present in about 75.8% of patients with MS.^3^^,^^4^ In this case, the absence of aortic dilation is a confounding factor that can make the diagnosis difficult.

The occurrence of ventricular fibrillation, in this specific case, could be due to several factors presented by the patient on admission, specifically the presence of MVP, QT interval prolongation, and documentation of asymmetric ventricular hypertrophy with scar burden on magnetic resonance imaging.

Arrhythmias are relatively common in MS, with atrial fibrillation being the most frequent (14.8% of patients).^5^ Some studies suggest that the occurrence of sudden cardiac death of presumably arrhythmic cause may occur in up to 4% of patients. However, these are series with a small number of patients, and the arrhythmic event was associated with greater left ventricular dilation.^6^^,^^7^ In a study including 12079 patients with MS, ventricular fibrillation accounts for 0.5% of all hospital admissions due to heart disease and 0.2% of all hospital admissions.^5^

In patients with MS, mitral valve prolapse and severe mitral regurgitation have a prevalence of 40% and 12%, respectively.^8^ Although the association between isolated MVP and ventricular arrhythmias is not consensual, studies suggest that the coexistence of moderate mitral regurgitation correlates with the presence of premature ventricular contractions and non-sustained ventricular tachycardia. However, it does not seem to predict the occurrence of severe arrhythmic events.^6^^,^^7^^,^^9^ In this patient, the marked reduction in the burden of ventricular arrhythmias after mitral valvuloplasty favors the hypothesis that the mitral valve disease was the primary factor leading to ventricular fibrillation.

QTc prolongation (>440 msec) may be present in up to 16-20% of patients with MS.^10^ Some studies have also demonstrated an association between left ventricular dilation, repolarization abnormalities, and the occurrence of ventricular arrhythmias.^6^^,^^7^^,^^10^ In this clinical case, left ventricular dilation was not documented, which suggests that long QT interval seems to be an independent predictor of ventricular arrhythmias. However, although the association with the presence of PVC and NSVT seems established, its association with more severe arrhythmic events does not seem to be well understood.^7^^,^^10^

Cardiomyopathy associated with MS is an increasingly recognized entity, but it is usually related to ventricular dilation and dysfunction.^3^ In this patient, the hypertrophic cardiomyopathy phenotype may be related to the mutation in the FNB1 gene. However, it is more likely that we are dealing with two separate conditions - hypertrophic cardiomyopathy and Marfan syndrome. The absence of a positive genetic test for HCM does not exclude the diagnosis, since the yield of the genetic test is only 30 to 40%.^11^ After reviewing the literature, we found only one case describing HCM concomitant with MS. Thus, this appears to be an extremely rare association that has not yet been previously studied.^12^ In the case described by Fujiseki et al., an asymmetrical hypertrophy of the interventricular septum was documented, with a septal-to-inferolateral wall thickness ratio of 2.5 (measured using M-mode). In the present case, there was a more pronounced asymmetry, with a septal-to-inferolateral wall thickness ratio of 3.2 (measured using MRI).

In this patient, the combination of these three factors (mitral prolapse with moderate regurgitation, long QT interval, and HCM) may have been decisive for the occurrence of ventricular fibrillation.

## Conclusion

This case reports an atypical presentation of MS with ventricular fibrillation, as opposed to aortic dilation and dissection. In a patient with ectopia lentis and a mutation in the FBN1 gene, a family history of sudden death supports the diagnosis of Marfan Syndrome in the absence of aortic root dilation. Although it is a subject with increasing attention, the evidence of malignant arrhythmias is still a gray area in MS. Left ventricular dilation and myocardial dysfunction may have a causal role, but these changes were not documented in this patient. Other alterations, namely the long QT and MVP with moderate regurgitation, could have contributed to the arrhythmic event. However, this patient also has HCM, which is an additional proarrhythmic factor, and whose association with MS seems to be extremely rare.
